# Response to: Comment on “Noninvasive Ventilation Weaning in Acute Hypercapnic Respiratory Failure due to COPD Exacerbation: A Real-Life Observational Study”

**DOI:** 10.1155/2019/4897045

**Published:** 2019-10-08

**Authors:** Paola Faverio, Anna Stainer, Federica De Giacomi, Grazia Messinesi, Valentina Paolini, Anna Monzani, Paolo Sioli, Irdi Memaj, Oriol Sibila, Paolo Mazzola, Alberto Pesci

**Affiliations:** ^1^School of Medicine and Surgery, University of Milan Bicocca, Respiratory Unit, San Gerardo Hospital, ASST Monza, Monza, Italy; ^2^Respiratory Department, Hospital de La Santa Creu I Sant Pau, Autonomous University of Barcelona and Biomedical Research Institute Sant Pau (IIB Sant Pau), Barcelona, Spain; ^3^School of Medicine and Surgery, University of Milan Bicocca, Acute Geriatrics Unit, San Gerardo Hospital, ASST Monza, Monza, Italy

We thank Karim and Esquinas [1] for their interesting comments.

Our study aimed to report a real-life experience and the personalized approach we used in the weaning phase [2]. We do agree with Karim and Esquinas that this approach with the number of days at each stage of weaning at physician discretion limits the generalizability of the results.

It is worth noticing, however, that most patients that completed weaning stayed at each stage the minimum time required by the protocol (1 day). The maximum time they spent in each weaning phase was 2 days. These data suggest that, although patients' evaluation and final decision is based on physicians' clinical judgment, having objective criteria and a weaning protocol to follow allows standardization of the treatment in most cases.

Regarding the 20 patients adapted to domiciliary noninvasive ventilation (NIV), the acute phase, meaning respiratory acidosis with respiratory distress, was solved in all cases. We prescribed domiciliary NIV in those COPD patients with persistent daytime hypercapnia after at least two episodes of acute hypercapnic respiratory failure (AHRF) requiring NIV in the prior year and/or comorbidities such as obstructive sleep apnea or obesity-hypoventilation syndrome. When considering the parameters used during ventilation, no difference was found between the inspiratory positive airway pressure (IPAP) and expiratory positive airway pressure (EPAP) used in the acute and chronic phase. From a clinical point of view, in some cases, both IPAP and EPAP were reduced 1 to 2 cmH_2_O from the acute to the chronic setting.

In regard to the risk of pneumothorax in patients with severe bullous emphysema, in our cohort, we only had one case out of 51 patients in whom NIV weaning was attempted; therefore, any further speculation is impossible. We think that, given the controversy of the subject, the need to prolong NIV application after the acute phase should be evaluated on a case-by-case basis and the risk of pneumothorax vs. the benefit of the prevention of AHRF recurrence must be carefully considered.

We agree with Karim and Esquinas that future studies are needed in order to better identify which subgroups of COPD patients with AHRF might benefit the most from the weaning process.
